# A Descriptive Study of the Clinical Presentation, Management, and Outcome of Horses with Acute Soft Tissue Trauma of the Tarsus and the Association with Synovial Involvement

**DOI:** 10.3390/ani12040524

**Published:** 2022-02-21

**Authors:** Taja Vajs, Omid Nekouei, Nora M. Biermann

**Affiliations:** 1 University Equine Hospital, Department for Companion Animals and Horses, University of Veterinary Medicine Vienna, Vienna 1210, Austria; nora.biermann@vetmeduni.ac.at; 2 Department of Infectious Diseases and Public Health, Jockey Club College of Veterinary Medicine and Life Sciences, City University of Hong Kong, Hong Kong 999077, China; omid.nekouei@cityu.edu.hk

**Keywords:** hock, equine orthopedic diseases, septic arthritis, antimicrobial treatment, wounds

## Abstract

**Simple Summary:**

The hock is commonly involved in traumatic injuries in horses and due to the large number of synovial structures located in the region, this frequently results in synovial infection which is a major and potentially life-threatening complication in horses. The aim of this retrospective study was to describe the clinical presentation, diagnostic procedures, management, and outcome of cases with acute soft tissue trauma to the hock and to identify the clinical features that were associated with synovial infection in these cases. This study found that increased synovial effusion, severe lameness on admission, and the persistence of lameness as well as certain wound locations were more commonly associated with a diagnosis of synovial infection. Advanced diagnostic modalities such as radiographs, ultrasonography, and measurements of inflammatory markers aided in establishing the correct diagnosis. The duration of systemic antimicrobial treatment was shorter than previously reported and many cases received local antimicrobial treatment at the site of injury, which may have improved its efficacy. At the time of discharge, while lameness was still present in some cases, the overall survival was excellent. This study describes the clinical features and treatment of these commonly encountered injuries, and this knowledge may benefit clinicians in the future.

**Abstract:**

The tarsus is one of the most common areas of traumatic injury with associated synovial involvement (SI) in horses. The aim of this retrospective study was to describe the clinical presentation, diagnostic procedures, management (emphasizing the type, duration, and route of antimicrobial administration), and outcome of cases with acute soft tissue trauma to the tarsal region. The presenting clinical features, the results of diagnostic modalities, and the initial response to therapy were assessed for their usefulness to predict SI. Medical records of 72 cases were included and SI was diagnosed in 34 cases (47.2%). Increased synovial effusion, lameness on admission (OR = 4.1; 95%CI 1.0–16.4), persistent lameness (OR = 5.7; 95%CI 1.8–17.9), increased blood SAA values (≥200 mg/L) from initial to second measurement (OR = 4.3; 95%CI 1.2–15.5), and wound location on the plantar/plantarolateral/plantaromedial compared to the lateral aspect of the tarsus (OR = 7.0; 95%CI 1.6–30.9) were associated with SI. Radiographs, ultrasonography, and the use of pressure testing when a wound was present proved to be useful in correctly diagnosing SI. The median duration of systemic antimicrobial administration was 8 (IQR: 5 to 9) days and most horses received local antimicrobial therapy. This study highlights several relevant clinical features and their association with SI and emphasizes the usefulness of local antimicrobial therapy in these cases.

## 1. Introduction

Horses are prone to orthopedic injuries due to their highly developed flight response [1]. There is little soft tissue cover in the distal limb and traumatic injuries resulting in penetration with the subsequent infection of synovial structures (SI) are common in horses [2]. The presence of SI and rapid, aggressive treatment are major determinants of overall prognosis and future soundness in these cases [3,4,5].

Varying information exists about the critical duration from the time synovial penetration occurs to the administration of the appropriate treatment [6,7,8,9,10,11,12]. It has been suggested that there is no difference in outcome whether endoscopic lavage is performed immediately after diagnosis, on an emergency basis, or the following day [11]. However, delaying treatment for more than thirty-six hours decreases the prognosis for a return to athletic function [12]. Ultimately, chronicity of infection is associated with an increased risk of permanent damage to associated structures such as articular cartilage, subchondral bone, tendons, and other soft tissue structures [13]. This is why traumatic injuries that involve synovial structures are still considered medical emergencies and a correct, non-delayed diagnosis essential to achieve the best outcome possible [6,8,11,14,15].

The correct diagnosis of SI can be complicated by difficulties in recognizing the extent of the injury and in identifying the precise location of the synovial structures in relation to the trauma. Particularly in regions with a large number and variety of synovial structures, clinicians require a thorough knowledge of all anatomic structures in the affected area. The tarsal region in equids creates a particular challenge as it encloses up to 12 synovial structures. These include not only joints but also several tendon sheaths and bursae [5,16,17]. Further, communications between these structures can vary between horses [17,18]. This makes it imperative for a clinician to link their knowledge with the presenting clinical features of the soft tissue injury for a correct diagnosis.

Treatment of SI in horses most commonly consists of a multi-modal approach, combining broad-spectrum systemic antimicrobials, endoscopic or needle lavage of the synovial structure with or without addition of local antimicrobial therapy, and analgesia provision [6,19]. It has been established that an endoscopic lavage is superior in fighting SI in adult equids compared to other lavage methods, particularly in cases of chronic infection and fibrin deposits within the synovial structure [9,10]. However, especially in cases of small synovial structures (e.g., subcutaneous calcaneal bursa, tarsometatarsal joint) or when there is extensive trauma to the surrounding soft tissue structures, other treatments (e.g., open debridement and lavage) may be considered more appropriate in selected cases [5].

Regardless of the choice of surgical treatment, broad-spectrum local and systemic antimicrobial therapy is a cornerstone of successful therapy. Different reports have described the use of systemic parental antimicrobial therapy for up to 10 days followed often by a prolonged course of systemic oral antimicrobials [2,4,5,8,15,20]. Guidelines on the duration and type of antimicrobial treatment in cases of SI have not been well established in the literature [2,4,5,6,8,15,19].

The aim of this retrospective study was to describe the clinical presentation, diagnosis, and management with an emphasis on the type, duration, and route of antimicrobial therapy, as well as the outcomes of cases with acute soft tissue trauma to the tarsal region. In addition, the results of diagnostic modalities and the initial response to therapy were assessed for their usefulness to predict SI.

## 2. Materials and Methods

The medical records of horses presented to the Equine University Hospital in Vienna between January 2016 and January 2021 with a soft tissue injury to the tarsal region following a history of acute trauma (<1 month prior to presentation) were reviewed [21]. Horses admitted with wounds, swelling, and/or acute lameness associated with the tarsal region were included. Patients with fractures, iatrogenic SI, lameness, or synovial effusion caused by chronic (>1-month clinical history) or developmental causes, SI due to generalized sepsis or prematurity, and horses with additional severe injuries that affected prognosis and treatment involving areas other than the tarsus were excluded.

Data collected from the medical records included: signalment (age, weight, breed, and sex), whether presentation occurred during or out of working hours, the reason for presentation (trauma, lameness, or swelling), the affected limb (left or right), if a referring veterinarian had examined the horse and the duration of clinical signs prior to presentation (in days). The presence of a wound, wound location (dorsal, dorsomedial, dorsolateral, plantar, plantaromedial, plantarolateral, medial, or lateral), wound size (estimated in mm^2^), wound exudate (present or absent), quality of wound exudate (purulent or serosanguinous), increased effusion of a synovial structure (present or absent), soft tissue swelling (none, around the wound, at the level of the tarsus, at the level of the tarsus and distally), and the presence and severity of lameness (scored 1–5/5 in walk [22]) were recorded upon arrival and on subsequent days of hospitalization. The findings of the diagnostic tests were recorded when performed and included: radiographic findings (no changes, changes unrelated to the presenting complaint, changes associated with a SI of the structure; for example, gas opacity proximal in the synovial structure, osteomyelitis, osteitis), ultrasonographic findings (no changes, changes unrelated to the presenting complaint, changes highly suspicious of penetration of a synovial structure; for example, communication with the wound, air visible within the synovial structure, hyperechogenicity of the synovial fluid), a pressure test of an adjacent synovial structure (not performed, negative, positive) and whether or not synoviocentesis was performed in one or more synovial structures, as well as synoviocentesis results based on cytology (normal, inflammatory, septic, questionable results). In addition, the cytology results of a synovial fluid analysis were recorded and comprised synovial total protein (TP; g/L), synovial total white blood cell count (WBC; <1.0 × 10^9^, 1.0–30.0 × 10^9^, >30.0 × 10^9^ cells/L), and neutrophil percentage (%) [23]. Measurements of blood serum amyloid A (SAA; mg/L) were also recorded and the most common measurement interval was every 48 h during the first seven to ten days of hospitalization.

Based on the clinical examination and the diagnostic test results, the presence or absence of SI was established for each case. Diagnosis of SI was based on synoviocentesis (cytology and/ or pressure testing) results, on diagnostic imaging (radiography and/ or ultrasound) results, and on a combination of both diagnostic approaches. In a few cases with a diagnosis of an infected subcutaneous/intertendinous bursa and lateral extensor tendon sheath, the basis for the diagnosis was not clearly stated in the medical records and the diagnosis was based on visual inspection and palpation during the initial clinical examination. The information collected in cases with SI is summarized in Table 1.

Lastly, outcome (survival to hospital discharge, euthanasia without attempting treatment, euthanasia following attempted treatment), cost of treatment (euro), duration of hospitalization (days) and whether lameness was present or absent at hospital discharge were recorded.

### Statistical Analysis

All statistical analyses were carried out using Stata v15.1 (StataCorp, College Station, TX 77845, USA). Frequency distributions of all collected data, e.g., horses signalment, physical examination findings upon admission, laboratory results, diagnoses, treatments, and outcomes of study cases were described and/or tabulated. For continuous variables, the median and interquartile range (IQR), and for categorical variables, the number of samples within each category were presented. For further analyses, the outcomes of study cases were dichotomized as the presence or absence of SI. Synoviocentesis results, blood SAA measurements at different time points, duration of antimicrobial use, duration of hospitalization, and treatment cost between cases with and without SI were compared using a two-sample T-test. In addition, treatment cost and duration of hospitalization were also assessed using the two-sample T-test for their association with the presence of wounds and cases requiring procedures under general anesthesia. Simple logistic regression was used to evaluate the potential univariable associations between the independent variables of interest including clinical features (soft tissue swelling, synovial effusion, presence of wounds, location of wounds, lameness upon presentation, lameness the following day, and lameness on discharge) as well as the results of diagnostic modalities (radiographs, ultrasonography, pressure testing) and the presence of SI (the dependent variable of interest). The results of the logistic regression models were presented as odds ratios (ORs), along with their respective 95% confidence intervals (CIs). For blood SAA values, the differences between the first and the second measurements, as well as the differences between the first and the last measurements obtained during hospitalization were calculated and their associations with SI were evaluated using a Chi-squared test. *p*-values of <0.05 were considered statistically significant.

## 3. Results

### 3.1. General Information

A total of 70 horses met the inclusion criteria and two of these horses presented twice during the study period, each for unrelated injuries and were therefore included as separate cases. Medians of age and weight at the time of presentation were 10 years (IQR 6 to 14 years) and 491 kg (IQR 373 to 568 kg), respectively. Mares accounted for 36/72 cases, geldings for 32/72 and stallions for 4/72 cases. The most common breeds were Warmblood horses (32/72), Quarter horse (9/72), Islandic horses (7/72), Thoroughbreds (6/72), and Standardbred (3/72). The remaining 15 cases comprised small numbers of different other breeds. The majority of cases (48/72) were admitted out of working hours and most cases were referred by a veterinarian (45/72) with a median duration since injury at admission of 1 day (IQR 0 to 5 days). The affected hindlimb was the right in 40/72 cases and the left in 32/72 cases.

### 3.2. Presenting Complaint and Clinical Features Upon Presentation

The majority of cases presented with a history of acute trauma (55/72); less common reasons for presentation were lameness localized to the tarsal region (10/72) and an acute swelling of the area (7/72).

SI was subsequently diagnosed in 34/72 cases, with the involvement of one synovial structure in 30/34 cases and the involvement of multiple synovial structures in 4/34 cases. Diagnosis of SI was based on synoviocentesis results in 10/34 cases, on diagnostic imaging (radiography and/ or ultrasound) results in 5/34 cases and on a combination of both diagnostic approaches in 16/34 cases. In three cases with a diagnosis of an infected subcutaneous bursa, lateral extensor tendon sheath or subcutaneous/intertendinous bursa, the basis for the diagnosis was not clearly stated in the medical records and the diagnosis was likely based on visual inspection and palpation during the initial clinical examination. SI was diagnosed in the calcaneal bursae (subcutaneous and/ or intertendinous and gastrocnemial) in 13/34 cases, in the tarsocrural joints in 10/34 cases, in the tarsal sheath in 5/34 cases, in the long and lateral digital extensor tendon sheaths in 5/34 cases, and in the tarsometatarsal joint and the distal intertarsal joint in one case.

A wound was present in 58/72 cases and the median wound size was 150 mm^2^ (IQR 40 to 275 mm^2^). Wounds were located in 17/58 cases on the lateral, in 7/58 cases medial, in 19/58 cases plantar, plantaromedial or plantarolateral, and in 15/58 cases dorsal, dorsomedial, or dorsolateral on the limb. The presence of wound exudate was reported in 23/58 cases and the quality of wound exudate was described as serous or bloody discharge in 12/23 cases and as purulent discharge in 11/23 cases. 

General soft tissue swelling was reported in 51/72 cases and was localized to one specific aspect of the tarsus or around the wound in 20/51 cases, to the entire tarsus in 22/51, and to the tarsus and distal limb in 9/51 of cases while increased synovial effusion was present in 40/72 cases. Lameness on admission was reported in 53/72 cases and was graded through walks as a 1/5 lameness in 14/5 cases, 2/5 in 13/53 cases, 3/5 in 12/53 cases, 4/5 in 13/53 and 5/5 in 1/53 cases.

### 3.3. Results of the Advanced Diagnostic Modalities

**Figure 1:**
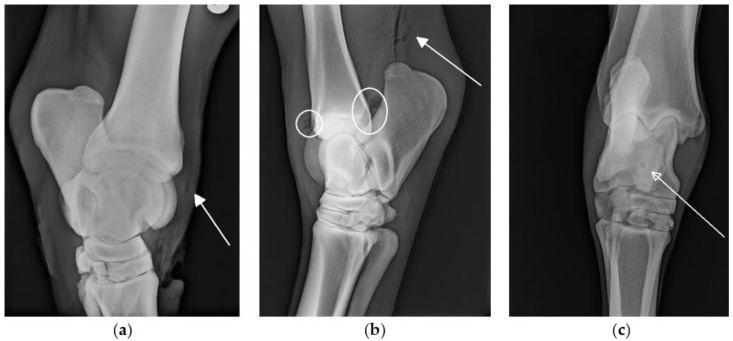
Radiographs of the tarsi with changes associated with SI (marked on the figures): Oblique latero-medial projection of the tarsus: (**a**) Air in the long digital extensor tendon sheath (arrow). (**b**) Latero-medial projection of the tarsus: air in the tarsocrural joint (circles) and subcutaneous air proximal to the calcaneous (arrow). (**c**) Dorsoplantar projection of the tarsus: round radiolucency at the level of the medial trochlea ridge of the talus (arrow) suggestive of osteomyelitis.

Radiographs were performed in 66/72 cases. In 10/66 cases, changes suggestive of SI, such as the presence of osteomyelitis or gas within a synovial structure (Figure 1) were seen on radiographs. In all 10 cases, a final diagnosis of synovial involvement was subsequently confirmed by additional diagnostic procedures (e.g., ultrasonography, synoviocentesis results). In 19/66 cases, synovial involvement was diagnosed based on other diagnostic procedures in the absence of radiographic changes to indicate SI. On the retrospective review of images, the radiographic identification of gas within bursae and tendon sheaths was subjectively more challenging than the radiographic identification of gas within the joints.

Ultrasonographic examination for the presence of SI was focused on findings including the presence or absence of air in the synovial structures, the direction of wound tracking in relation to the synovial structures, and alterations in the echogenicity of the synovial fluid or the appearance of the synovial structure lining. Ultrasonographic findings were recorded in 46/72 cases. Changes highly suggestive of SI were reported on ultrasonographic examination in 18/46 cases and of these, 16 of these cases were subsequently diagnosed with SI. No changes indicating SI were reported on ultrasonographic examination in 28/46 cases and in seven of these cases SI was diagnosed using other diagnostic modalities.

Synoviocentesis of one or more structures was performed in 58/72 cases and the results of the cytological analysis of the synovial fluid samples when reported are displayed in Table 2. Specifically, the TCJ was sampled in 33/58 cases, the tarsal sheath was sampled in 9/58 cases, the intertendinous calcaneal bursa was sampled in 9/58 cases, followed by the subcutaneous calcaneal bursa in 4/58, the tendon sheath of the long digital extensor tendon in 2/58 cases, and the tarsometatarsal joint in 1/58 case.

Further, when a wound was present in close proximity to a synovial structure, pressure testing by the administration of a sterile physiological solution was performed in 24 cases and proved the communication between a synovial structure and the wound in 10/24 cases and the absence of communication in 14/24 cases; 2/24 of these cases were subsequently diagnosed with SI based on a cytological examination of the synovial fluid.

**Figure 2:**
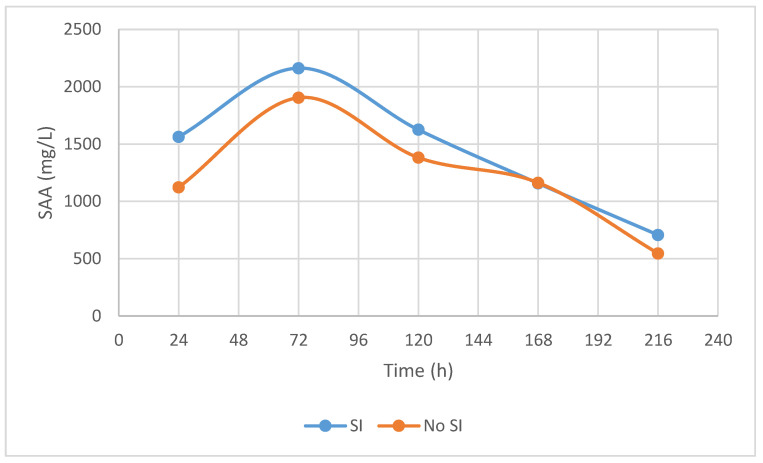
Trends of mean blood SAA values (mg/L) in horses with (SI) and without (No SI) synovial involvement during hospitalization.

Blood SAA was measured in 62/72 cases and results are displayed in Figure 2.

### 3.4. Factors Associated with SI

**Figure 3:**
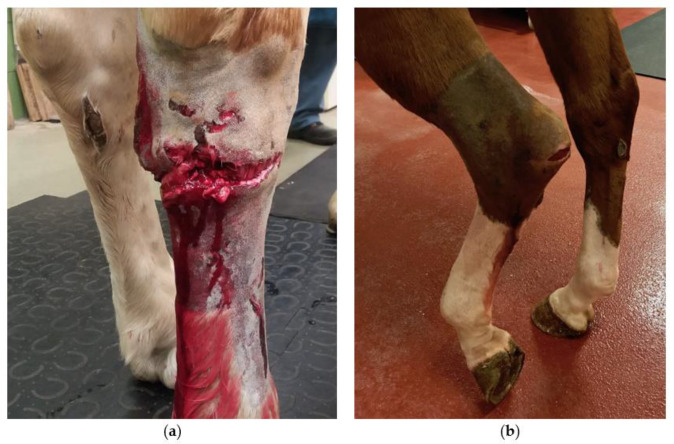
Examples of wounds on the plantar aspect of the tarsus with associated SI. (**a**) Wound at the plantar aspect of the right tarsus approximately 10 cm distal to the tuber calcanei with involvement of the tarsometatarsal joint. (**b**) Wound at the plantar aspect of the left tarsus with SI of the subcutaneous and intertendinous calcaneal bursae.

The presence of a wound on admission did not change the odds of SI (*p* = 0.817), as synovial infection was diagnosed in 27/58 cases where a wound was present and in 7/14 cases where no wound was present on admission. However, when a wound was present, the location of the wound was significantly associated with the presence of SI (*p* = 0.049). Wounds located on the plantar, plantarolateral, or plantaromedial aspect of the limb were more likely to be associated with SI (OR = 7.0; 95%CI 1.6–30.9; *p* = 0.010) compared to the referent of wounds located on the lateral aspect of the limb (Figure 3). The presence of synovial effusion was associated with SI (OR = 8.6, 95%CI 1.0–76.1; *p* = 0.028). The presence of lameness at admission was associated with a diagnosis of SI (OR = 4.1, 95%CI 1.0–16.4; *p* = 0.040). In particular, horses with severe lameness upon presentation were 5.3 times more likely to have SI compared to horses with no or very mild degrees of lameness (OR = 5.3, 95%CI 1.3–25.5; *p* = 0.023). These horses were also more likely to still show some degree of lameness the day after admission (OR = 5.7, 95% CI 1.8–17.9; *p* = 0.003). While blood SAA measurements did not differ at any time point significantly between horses with and without SI, an increase of ≥200 mg/L in blood SAA from the initial to the second blood SAA measurement was associated with SI (OR = 4.3, 95% CI 1.2–15.5; *p* = 0.023). In addition, horses with SI had a larger decrease (>1000 mg/L compared to <1000 mg/L) in SAA values from their initial blood SAA sample to the last measurement (*p* = 0.046) (Figure 2).

### 3.5. Treatment

An endoscopically guided lavage of a synovial structure under general anesthesia was performed in approximately one third of the cases (25/72), with one of those cases undergoing an endoscopic lavage of the joint due to hemarthrosis without a concurrent septic process. Of these cases, 10 cases underwent endoscopic lavage under general anesthesia once, six cases twice, two cases three times, three cases four times, two cases five times, and one case six times. In 6/25 cases a lavage was performed using standing sedation in addition to procedures under general anesthesia. Wound management was at the discretion of the primary treating clinician and included the primary closure of a wound with or without a placement a drain, wound debridement, lavage and bandaging, stabilization by a bandage/splint construct or medical therapy only. 

One quarter of cases (9/34) with confirmed SI were treated by a standing lavage via needle, followed by the primary closure of the wound if a wound was present. In one additional case a wound involving the subcutaneous calcaneal bursa was left open to heal by second intention after the wound and bursa were debrided and lavaged.

#### Antimicrobial Use

Systemic antimicrobial treatment was administered in 69/72 cases and the total median duration of systemic antimicrobial administration was 8 days (IQR 5 to 9 days). SI was associated with a significantly increased duration of systemic antimicrobial administration (9.32 ± 4.46 days vs. 5.4 ± 1.97 days; *p* < 0.001) and with a significant increase in the use of regional antimicrobial delivery (30/34 vs. 26/38; *p* = 0.043). Intrasynovial administration was frequently performed at the same time when a synoviocentesis was performed (53/58 cases). While wounds were present in 10/12 cases where a second antimicrobial regimen was administered, the presence of a wound in the area did not change the total duration of antimicrobial administration (*p* = 0.550). However, when a wound and SI were present, the total duration of antimicrobial administration was longer than when only a wound was present (11.0 ± 6.4 days vs. 5.4 ± 2.2 days; *p* < 0.001).

When treated with systemic antimicrobials, horses most commonly initially received a combination of Penicillin (30,000 IU/kg IV every 6 h) and Gentamicin (6.6 mg/kg IV every 24 h) (62/69). All other cases initially received Trimethoprim-Sulfadiazine (30 mg/kg PO every 12 h) (7/69). The median duration of the systemic administration of the initial antimicrobial selection was 7 days (IQR 4.5 to 9 days).

After the initial antimicrobial treatment, 12/69 cases received a second course of systemic antimicrobials with a median duration of 4 days (IQR 2.5 to 6 days). Here, the majority of cases received Trimethoprim-Sulfadiazine (30 mg/kg PO every 12 h) (7/12), followed by Doxycycline (10 mg/kg PO every 12 h) in 3/13 of cases and one case each received a combination of Penicillin (30,000 IU/kg IV every 6 h) and either Gentamicin (6.6 mg/kg IV every 24 h) or Marbofloxacin (2 mg/kg IV every 24 h).

Regional antimicrobial (intrasynovial injections or intravenous regional limb perfusion) therapy was administered in 56/72 cases. In 52/56 cases antimicrobials were administered via intrasynovial administration and in 11/56 cases via regional limb perfusion. A combination of both regional antimicrobial therapies was performed in only 7/56 cases. An aminoglycoside, either Gentamicin (250–2000 mg per synovial structure or treatment) or Amikacin (500–2000 mg per synovial structure or treatment) was administered by intra-synovial injection (47/52) and via regional limb perfusion (10/11). In five cases with persistent SI, Vacomycin (Gycopeptide; 1000 mg intra-synovial) was used. Of those, a bacterial culture from synovia was submitted in three cases and antibiotic sensitivity testing was performed, but sensitivity to Vancomycin was not tested.

### 3.6. Outcome

The majority of horses with soft tissue trauma to the tarsus survived to hospital discharge (70/72). The two non-survivors were both diagnosed with SI and this resulted in a slightly lower survival rate for patients with SI (32/34). One of these cases was euthanized following diagnosis without treatment and the other horse was euthanized after treatment was attempted but infection persisted. Two horses re-presented for the recurrence of clinical signs. One of these cases exhibited severe lameness 6 days following hospital discharge and was diagnosed with persistent SI of the intertendinous bursa. Based on the owners’ request, human euthanasia was performed at the time without further attempted treatment. The other case presented with a persistent non-healing wound, swelling around the tarsus, fever, and a subdued demeanor, and was subsequently diagnosed with SI of the lateral extensor tendon sheath, with further progression to SI of the tarsal sheath and subcutaneous calcaneal bursa. The horse was treated and again discharged from hospital. In addition, one horse was discharged, prior to the confirmed resolution of infection of an intertendineous bursa, based on the owners’ request and on the follow up six months later, the owners reported that the horse showed no lameness in their walk.

Mild to moderate lameness (grade 1–3/5) at the time of discharge was still present in 13/70 horses and was not associated with a diagnosis of SI (*p* = 0.084).

The median duration of hospitalization was 13 days (IQR 6.5 to 23 days) and neither the presence of SI nor wounds increased the time spent in hospital significantly. The median cost of treatment for all cases of soft tissue injury to the tarsus was EUR 1984 (EUR IQR 1263 to 3840) and the cost was significantly higher for cases with SI (EUR 4792 ± EUR 3296 vs. EUR 1598 ± EUR 944; *p* < 0.001) mostly based on the need for one or multiple endoscopic lavages under general anesthesia, as the median cost for these cases was EUR 4840 (EUR IQR 3188 to 8092) compared with EUR 1471 (EUR IQR 1152 to 2009) (*p* < 0.001).

## 4. Discussion

This study has described several clinical features of cases with soft tissue trauma to the tarsus region that have not been previously described in the literature. Nearly half of all cases were diagnosed with SI and while there were a large number of synovial structures located at the tarsus, the most commonly affected were bursae on the plantar aspect of the limb rather than the larger joint compartments of the tarsal joints dorsally.

Several studies have emphasized the need for the prompt diagnosis and treatment of SI in horses [6,8,11,14,15]. Certain clinical features, e.g., the severity of lameness on admission or increased synovial effusion, are commonly identified in horses with SI and help to direct additional diagnostic efforts [24,25]. The lack of other ubiquitous clinical features associated with SI, however, emphasize the need for a thorough and complete diagnostic investigation. In particular, the absence of a wound does not preclude a diagnosis of SI in this study. Since horses with a history of iatrogenic SI were excluded, very small penetrating injuries that had closed up at the time of examination were the most likely explanation for SI in these horses. However, we cannot rule out that some of these cases could have been iatrogenic in nature and the owners may have failed to disclose this information. Cases without a wound or with no obvious reason for SI have previously also been described in literature as idiopathic or due to hematogenous spread [6,11,20,24].

When a wound was present, it was most commonly found on the lateral aspect followed by the plantar aspects of the limb and, accordingly, the plantar structures were more likely to be affected by SI. In this aspect of the tarsus, the calcaneal bursae, the tarsal sheath and the plantar joint pouches of the tarsocrural joint and smaller distal tarsal joints are located very close to the skin surface [16] and may be more easily penetrated. More specifically, the calcaneal bursae were the most common structures to show signs of SI and their location and resulting exposure at the tuber calcanei are likely to account for these results. It has been previously reported that the diagnosis of septic bursitis in the calcaneal region can be challenging, resulting in a delayed diagnosis in some cases [5,26]. Our clinical impression and findings in this study would support this, since many cases presented for trauma and lameness located to the tarsus, but not for suspected SI. One of the cases in this study was initially treated only for a wound and re-presented shortly after discharge with several septic synovial structures, which may have already been present during the initial hospital stay, despite diagnostic investigation in a referral center, highlighting the diagnostic challenge in these cases.

The low number of cases involving distal intertarsal and tarsometatarsal joints may be explained by the exclusion of cases with bony abnormalities due to trauma, e.g., proximal splint bone fractures. The inclusion of these cases would have required taking fracture configuration, treatment, and prognosis aside from synovial infection into consideration, which would have been beyond the scope of this study. 

Routine diagnosis of SI is based on a combination of history, clinical signs, diagnostic imaging, synovial fluid analysis, and blood parameters. If a sample of the synovial fluid can be obtained, changes in synovial fluid parameters are most commonly used for initial diagnosis and for the determination of a treatment plan [2,24]. Particularly when small synovial structures are involved, obtaining a synovial fluid sample can be difficult [5,26] and additional diagnostic modalities may be required to obtain a diagnosis. 

Diagnostic imaging, primarily radiography and ultrasonography, was performed in the majority of cases and assisted in correctly diagnosing SI but were not reliable methods to exclude synovial penetration entirely. Changes seen on radiographs when SI is detected later in the disease process include joint space collapse, osteitis, osteomyelitis, and calcification of periarticular tissues [27]. These changes are not usually seen radiographically in adult horses until at least 14 days post injury [5] but baseline radiographs can help to distinguish possible signs of progressive infection from the pre-existing pathology [27]. Faster progression of radiographic changes has been described in association with certain bacterial isolates, e.g., *Staphylococcus aureus,* and may be associated with a decreased prognosis [28].

Ultrasound has previously been reported as a highly useful tool to assess soft tissue structures, in particular bursae and tendon sheaths [5,29,30]. In this study, it was used to assess the volume and quality of synovial fluid within the synovial structures and to guide the approach for synoviocentesis. An altered echogenicity of the synovial fluid was not consistently associated with SI, but where there was clear evidence of communication between wounds and synovial structures on a systematic ultrasound examination then this was helpful is establishing a diagnosis of SI. Particularly in cases with the involvement of small synovial structures, e.g., extensor tendon sheath and calcaneal bursae, diagnosis of SI was sometimes solely based on results of this modality. In a small subset of cases (3), the diagnostic modality that identified SI was not clearly stated in the medical records. Based on the available information, visual inspection and palpation during initial clinical examination were likely conclusive and highlight the need for a thorough knowledge of anatomic structures and their location in this area.

SAA is an acute phase protein with values increasing rapidly in the systemic circulation in response to inflammation and infection [31]. It has been reported to increase in cases of SI [31,32], which was also seen in our study. Total blood SAA concentration did not differ between horses with or without SI at any time point, with an increase of at least 200 mg/L between the first and second measurement indicative of SI.

Multimodal approaches, consisting of a combination of synovial lavage, pain management, and broad-spectrum antimicrobials administered systemically and locally are commonly performed to treat synovial infection in horses [2,10]. In our study, general anesthesia was only required in cases with synovial pathologies, either SI or hemarthrosis. In approximately two thirds of cases, primarily when larger synovial structures were involved, endoscopic lavage and debridement were performed initially. In more than half of these cases, more than one surgery was required during hospitalization. 

When wounds were present, the primary closure of the wound or healing by second intention were elected depending on the type of wound, its location, and the degree of contamination. Particularly when wounds communicated with small synovial structures such as extensor tendon sheaths or subcutaneous calcaneal bursa, lavage, and primary wound closure after debridement in the standing sedated animal were elected in many cases rather than endoscopic lavage.

In some cases of persistent SI, where multiple lavages were required, both lavages under general anesthesia and in standing sedated horses, were used to decrease the treatment cost and to avoid the risks of general anesthesia. 

Systemic antimicrobial therapy with broad-spectrum antimicrobials, for example a combination of a beta-lactam and an aminoglycoside (e.g., Penicillin and Gentamicin or Amikacin), is widely accepted for the treatment of SI [2,4,5,8]. Except in a small number of cases, classes of antimicrobials were used here that are not considered reserve or critically important antimicrobials in human medicine; however, the clinical rationale particularly for the use of a last resort antimicrobial such as Vancomycin was not clearly stated in the medical records. This highlights the need for critical assessment and documentation in cases where the use of these medications is considered. The duration of systemic parental antimicrobial therapy for up to 10 days followed often by a prolonged course of systemic oral antimicrobials has previously been reported [2,4,5,8,15,20]. Average duration of total antimicrobial administration in our study was eight days. These also included cases where a second course of oral systemic antimicrobials was administered, which was not required in the majority of SI cases. Therefore, both the total duration of systemic antimicrobial administration, as well as the use of a second course of systemic antimicrobials, was lower here than previously reported. 

Further, some studies have reported that systemic antimicrobial treatment continued after hospital discharge [2,5,8]. In contrast to these studies, very few horses in this study were discharged with the recommendation to continue antimicrobial therapy at home. These findings might be partly due to the frequent use of local administration of antimicrobials, either intrasynovial or via regional limb perfusion in our study. Local delivery of antimicrobials is increasingly used and is effective in achieving high concentrations in the specific region, while decreasing the treatment cost and the potential side effects of systemic administration [33,34,35,36]. There has been considerable research on regional limb perfusion protocols in experimental settings [37,38,39,40] but very limited reports on frequency and efficacy of local antimicrobial administration in naturally occurring traumatic injuries and in SI in horses [39]. It appears that the ideal protocol has not yet been established and doses used in clinical practice still vary widely [41]. This is also shown in the results of our study, where dosages of both intra-synovial injections and regional limb perfusion protocol varied widely. Due to the large variety of cases and treatments described in this study, the effect of local antimicrobial administration regarding clinical outcomes and the shortened duration of systemic administration of antimicrobials cannot be accurately determined. However, the combination of findings in this study, namely the shorter duration of systemic antimicrobial administration, the reduced systemic antimicrobial administration after discharge, and comparable or improved survival rates, is encouraging [4,8,10,20]. Further work to standardize treatment protocols and to determine the efficacy of local antimicrobial administration in naturally occurring SI and traumatic injuries could benefit case outcomes and facilitate the reduced use of systemic antimicrobials in these cases.

Antimicrobial use, particularly systemic, in cases where deeper structures are not involved in an area that requires further research. Currently, the majority of traumatic injuries in equine medicine appear to be treated with antimicrobials and clear guidelines are missing. In human medicine, recommendations on systemic antimicrobial treatment in these cases is highly dependent on the degree of contamination and tissue damage [42,43]. Administration of antimicrobials after traumatic injuries of distal limbs in horses has been justified by their inherent environmental contamination. With the continued evolution of antimicrobial resistance, these practices need to be critically assessed in injuries where deeper structures are not affected. Thorough cleaning and debridement combined with or without local delivery of antimicrobials via regional limb perfusion could be considered rather than use of systemic antimicrobials.

There are limitations to synovial cytology and the values change for a variety of reasons (i.e., following endoscopic lavage and intrasynovial treatment [44,45,46,47]). To determine the proper endpoints of treatment as well as treatment success is challenging. In our hospital, serial blood SAA measurements were used, in addition to clinical response and synoviocentesis results, to monitor treatment success. In this study, a larger decrease in blood SAA values was seen with SI compared to horses without SI from first to last sample taken during hospitalization, consistent with previous reports that treatment success in cases of SI can be monitored using blood SAA values [48]. Clinically, horses with SI were still more commonly lame the day after admission than those without. This could be a result of more aggressive treatment via the synovial lavage under general anesthesia, but it may also indicate that lameness generally persists longer in these cases. However, there was no difference in lameness between horses with and without SI at the time of discharge.

Overall, survival to hospital discharge was excellent although some degree of lameness was still present in about twenty percent of these horses. Unfortunately, we were unable to conduct a consistent follow-up to determine long-term prognosis and soundness, but these results suggest a positive outcome in the majority of cases.

This study has several limitations. Particularly, the retrospective nature of the study and the resulting incomplete information especially on the clinical description of injuries and the details of treatment, as well as a lack of follow up beyond hospital discharge have to be considered when interpreting the results. Another limitation is the lack of bacterial culture and sensitivity data as there were only a very small number of samples submitted and therefore the results were not included. Bacterial cultures and the sensitivity assessment of synovial fluid samples are considered as the gold standard in diagnosing synovial infection. Previous studies have showed that these are positive in only 22–75% of cases and the results generally take several days during which the treatment is already established [3,8]. Historically, positive culture results have very been rarely obtained in our institution, which has resulted in a reluctance by clinicians to submit samples in cases of SI. In order to improve best practices of antimicrobial stewardship, this is an area with potential for improvement and will be addressed in clinical rounds in the future.

## 5. Conclusions

In summary, in about half of the cases of soft tissue trauma to the tarsus, one or more synovial structures were involved, and SI was most common in the plantar region. Additionally, clinicians have to be aware that the absence of an obvious wound in this area does not rule out SI in these cases.

Advanced diagnostic modalities proved useful to establish a correct diagnosis of SI and the use of consecutive SAA measurements is helpful to ascertain a diagnosis as well as to monitor the response to treatment. Lastly, while no definitive causal relationship between the use of local antimicrobial treatments and shortened total duration of systemic antimicrobial therapy could be established in this study, the excellent prognosis of horses presented for soft tissue trauma to the tarsus may support local antimicrobial use in future cases. 

## Figures and Tables

**Table 1:** Information obtained in cases with SI.

Synovial structure involved:	Talocrural joint and proximal intertarsal joint		
	Tarsal sheath		
	Extensor tendon sheaths		
	Subcutaneous and/or intertendinous and gastrocnemial calcaneal bursae		
	Talocrural joint and tarsal sheath		
	Tarsometatarsal joint and/or distal intertarsal joint		
Treatment:	Type of treatment:	Wound debridement and lavage	
		Primary wound closure	
		Endoscopic lavage	
	Systemic antimicrobial therapy:	Penicillin G 30,000 IU/kg IV every 6 h	
		Gentamicin 6.6 mg/kg IV every 24 h	
		Doxycycline 10 mg/kg PO every 12 h	
		Trimethoprim-sulfonamide 30 mg/kg PO every 12 h	
		Marbofloxacin 2 mg/kg IV every 24 h	
	Local antimicrobial therapy:	Intrasynovial therapy:	Amikacin 500–2000 mg per synovial structure
			Gentamicin 250–2000 mg per synovial structure
			Vankomycin 1000 mg per synovial structure
		Combination of intravenous regional limb perfusion and intrasynovial therapy:	Amikacin
			Gentamicin
			Penicillin
			Use of different antimicrobials through the treatment

**Table 2:** Synoviocentesis results in cases with and without synovial involvement (SI) and their association with SI.

Variable		No Synovial Involvement			Synovial Involvement			*p*-Value **
	N *	n	Mean	SD	n	Mean	SD	
TP (g/dL)	25	11	25.6	16.9	14	47.6	15.6	0.003
WBC (cells/L)	29	13	13,210.0	30,231.0	16	53,346.9	45,934.9	0.012
Nf (%)	41	21	30.1	24.0	20	89.6	19.6	<0.001

* N: total number of cases where the parameter was recorded. n: number of cases with or without synovial involvement of cases where the parameter was recorded. TP: synovial total protein (g/dL), WBC: synovial total white blood cell count (cells/L), Nf: synovial neutrophil percentage (%). SD: standard deviation, *p*-value > 0.05 were considered significant, ** *p*-values are from two-sample T-tests.

## Data Availability

Not applicable.
